# Transvaginal natural orifice endoscopic surgery for myomectomy: Can it be a conventional surgery?

**DOI:** 10.3389/fsurg.2022.1013918

**Published:** 2022-11-04

**Authors:** Qiannan Hou, Xin Li, Lu Huang, Ying Xiong, Dan Feng, Qiang Zhang, Xiaoyan Zeng, Yang Yang, Tianjiao Liu, Yalan Li, Yonghong Lin, Li He

**Affiliations:** ^1^ Chengdu Women’s and Children’s Central Hospital, School of Medicine, University of Electronic Science and Technology of China, Chengdu, China; ^2^ The Fourth People’s Hospital of Chengdu, School of Medicine, University of Electronic Science and Technology of China, Chengdu, China

**Keywords:** transvaginal natural orifice transluminal endoscopic surgery, minimally invasive surgery, myomectomy, myoma, retrospective study

## Abstract

**Introduction:**

As a new minimally invasive surgery, transvaginal natural orifice transluminal endoscopic surgery (vNOTES) has been proved to be suitable for the treatment of a variety of gynecological benign diseases. However, compared with other minimally invasive surgeries that have been widely used, such as conventional multiport laparoscopy and transumbilical laparoendoscopic single-site surgery (LESS), their advantages and disadvantages and how to choose are still unknown. The purpose of our study is to compare the advantages and disadvantages of the three minimally invasive surgeries in myomectomy and to provide theoretical basis for the wider development of vNOTES surgery.

**Material and methods:**

This retrospective study included 282 patients at our hospital who underwent laparoscopic myomectomy from May 2021 to March 2022. Based on the surgical approach, patients were classified into multiport, transumbilical LESS, and vNOTES groups. The patients’ demographic characteristics and follow-up data were collected during the perioperative period and at 1 month postoperatively.

**Results:**

Among the three procedures, vNOTES had the shortest anal exhaust time but also the highest postoperative infection rate. Multiple linear regression analysis showed that the operative time increased by 3.5 min for each 1 cm increase in myoma, and intraoperative bleeding increased by approximately 12 ml. The average duration of single pores increased by 25 min compared to that of multiports, and the operative duration increased by 10.48 min for each degree of adhesion.

**Conclusions:**

For gynecologists who have mastered vNOTES, this procedure has the same efficacy and safety as the two existing minimally invasive surgeries in myomectomy, but it shows obvious advantages in postoperative recovery.

## Introduction

Fibroid is one of the most common gynecologic conditions, but it does not occur before puberty and its frequency decreases with menopause (1, 2). They are diagnosed in 20%–25% of women of reproductive age and in 30%–40% of women older than 40 years (3–5). They originate from the clonal expansion of a smooth muscle cell stimulated by estrogen and progesterone (6, 7). Myomas may grow asymptomatically. When symptoms occur, abnormal uterine bleeding, urinary or bowel dysfunction, infertility, and abdominal pain are mainly reported (8–10). Thus, women with this disease have high risks of developing physical and emotional distress, which can strongly impact their life (11).

Several treatments for myoma are available, such as medical treatment, surgery, high intensity focused ultrasound (HIFU), and interventional radiology (12–15). Among them, myomectomy is the gold standard surgical method when fertility sparing and refusal of hysterectomy are demanded (8, 16). Over the past decade, minimally invasive surgery has become the popular goal of surgeons. Younger patients further pursue better cosmetic effect, resulting smaller-scar or scarless surgery. Hence, needlescopic-assisted laparoscopy, transumbilical laparoendoscopic single-site surgery (TU-LESS), and vaginal natural orifice transluminal endoscopic surgery (vNOTES) came into being (17). For myomectomy, the last two operations hide the incisions in the belly button and the vagina to become scarless (18–20). However, few previous studies had compared their characteristics with conventional laparoscopic surgery for myomectomy.

Since Baekelandt's study in 2018 showed that vNOTES could be used for removal of uterine fibroids (21), a large number of previous studies have confirmed the possibility of vNOTES myomectomy (20, 22). However, due to the anatomical specificity of the vNOTES approach, the risk of injury to the adjacent organs (rectum and bladder) is greater. At the same time, for the limitation of visual field, it is more difficult to deal with the lesions in the fundus uterus and the two lateral walls. In gynecological benign surgery, incomplete suture and penetration of uterine cavity have made myomectomy with a high rate of postoperative complications (23–25). Therefore, under the proven feasibility of vNOTES surgery, it is necessary to compare the information of perioperative periods of prevalent surgical methods to confirm the safety of vNOTES surgery.

Therefore, in the present study, we investigate the perioperative data of vNOTES in myomectomy and compare them with mutiport and single-port laparoscopic surgery. According to result of this research, the optimum surgical approach could be chosen by gynecologists with patients’ own characteristics and hospital conditions. In addition, we provide more cautions for other surgeons when performing this novel surgery.

## Materials and methods

### Study design and participants

**Figure 1:**
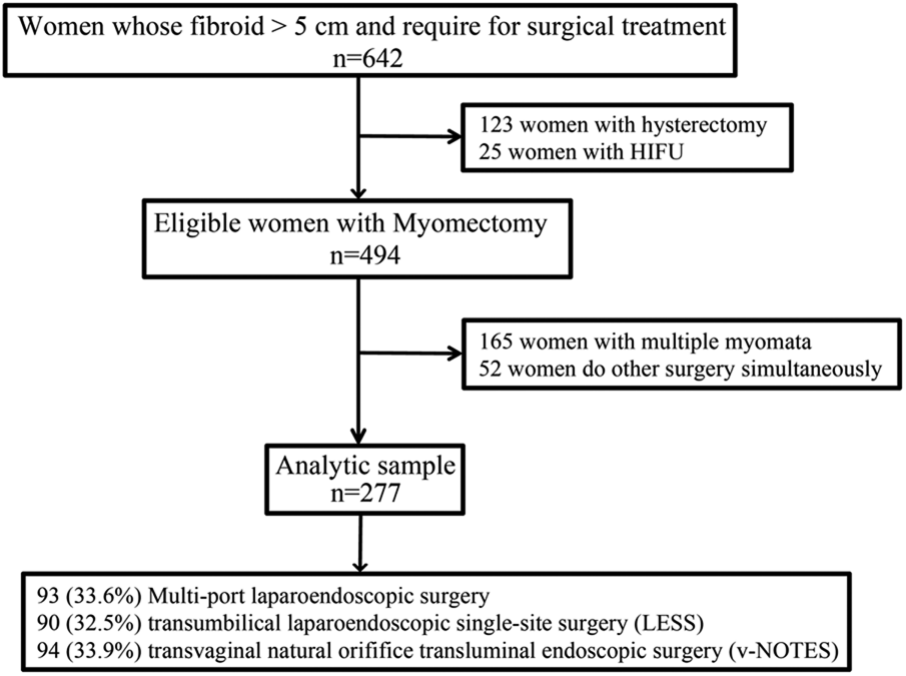
The selection process for this study.

The present study was embedded in the Longitudinal Vaginal Natural Orifice Transluminal Endoscopic Surgery Study (LovNOTESS), an ongoing gynecological minimally invasive surgery study conducted in Chengdu, aiming to determine the short- and long-term complications, as well as the potential effects of vNOTES on patients’ sexual function, pregnancy, and vaginal delivery (Chinese Clinical Trial Registry ChiCTR2100053483), approved by the Ethics Committee of the Chengdu Women's and Children's Central Hospital (No. 202130). During the study period, the total number of laparoscopic surgery per year ranged between 3,500 and 4,000. Written informed consent was obtained from all participants. This subgroup study only included the retrospective clinical data of all patients who were diagnosed with uterine fibroid (symptomatic subserosal fibroids and intramural fibroids, tumor size >5 cm) and came to seek a surgical solution between May 2021 and April 2022 at the Chengdu Women's and Children's Central Hospital. On further analysis, patients who had multiple uterine fibroids and underwent hysterectomy were excluded. According to the postoperation pathological reports, patients with adenomyoma were also eliminated. Then, in terms of different operative approaches, all the participants were classified into the multiport group, TU-LESS group, and vNOTES group (Figure 1).

### Data collection

The information of all patients were collected from the hospital database, including patient's age, body mass index (BMI), maximum diameter and location of the myoma, history of previous gravidity and abdominal surgery, operative position, total operation time (from skin incision to closure), blood loss [use Subjective Visual Quantification (26)], simultaneous surgery, intraoperative complications (injuries to the bladder, bowel, and vessels), conversion to another surgical procedure, postoperative serum hemoglobin drop, time of flatus after surgery, postoperative fever [any oral temperature of 38.0°C or more occurring 24 or more hours postoperatively (27)], hospital stay, and postoperative complication [scaled using the Clavien–Dindo complication rating (28)] during the 1-month period after surgery. All patients underwent outpatient review 1 month after surgery to check postoperative recovery and complete clinical data.

### Standard operating procedures of vNOTES

#### Preoperation

For bowel preparation, all the patients were given sodium phosphate oral solution the day before surgery. In addition, patients in vNOTES group were given iodophor vaginal scrubbing twice a day.

All the surgeries were performed under general anesthesia. For both multiport and TU-LESS myomectomy procedures, the patients were placed in the dorsal lithotomy position. For the vNOTES group, some patients in the subgroup of posterior uterine myoma were placed in the prone position (*n* = 35). Cefmetazole 1 g was given intravenously 30 min before vNOTES surgery for bacterial infection prophylaxis. The vagina and perineal area were sterilized with iodophor and a Foley catheter was indwelled for all patients.

#### Intraoperation

In the multiport group, we used the four ports technique, including two 12 mm trocars (for the umbilical port and one of lateral ancillary port) and two ports using 5-mm trocars. When undergoing myoma morcellation, an electric motorized morcellator (ROTOCUT G1 Morcellator; Karl Storz Endoskope, Tuttlingen, Germany) was placed through the 12 mm lateral ancillary port. Morcellation was performed according to the standard technique (7). In the TU-LESS group, a 2 cm incision was made at the umbilicus. Then, a multiple-instrument access port (Beijing Aerospace Kadi Technology Development Institute, HK-TH-60.4TY) was inserted through the incision. However, in the vNOTES group, the access paths of the pelvic cavity had various ways (through anterior or posterior fornix), in accordance with the location of the myoma. Operating platform was still built by a multiple-instrument access port.

The next steps were same and were followed in all groups: Pneumoperitoneum was created with up to 14 mmHg of CO_2_ insufflation and a 10-mm 30° rigid laparoscope (Karl Storz GmbH & Co. KG, Tuttlingen, Germany) was used for visualization. Initially, the location of the fibroid was identified. Then, Pituitrin 6 u was injected into the myometrium and the protruding site was incised by a unipolar hook. After enucleation of the myoma, the uterine wound was closed using 0# barbed absorbable suture (V-LOC 180 Absorbable Wound Closure Suture by Medtronic USA). The mass was bagged and removed from the only incision in the TU-LESS group and the vNOTES group. However, in the multiports group, morcellation was performed according to the standard technique through the 12 mm lateral ancillary port.

If intraoperative injury of large vessels or important organs and bleeding >800 ml occurred, the surgical method will be changed. vNOTES is generally converted to transabdominal single-port laparoscopic surgery, and single-port laparoscopic surgery is converted to multiport surgery. Immediate conversion to open surgery should be made if there is a life-threatening vascular injury.

Peritoneal adhesions were evaluated and classified according to Nair's scoring system. Adhesion is divided into four degrees according to the adhesion between two viscera and viscera and abdominal wall (29).

The abdominal and vaginal wounds were closed with a 2-0 absorbed suture and a 2-0 barbed absorbable suture, respectively. In the multiports group, a drainage tube was implanted in some cases. The drainage tube was not placed conventionally in TU-LESS and vNOTES groups.

### Statistical analysis

All statistical analyses were performed with SPSS version 25.0 (IBM, Armonk, NY, United States). Continuous variables are presented as mean and standard deviation and were analyzed by Student's *t*-test, LSD Student's *t*-test, one-way analysis of variance, or the nonparametric test. Categorical variables are presented as the count and percentage and were analyzed by the chi-squared or Fisher's exact test. For statistical accuracy, cases with large discrete size (intraoperative bleeding >500 ml) were excluded before data analysis, which will be discussed in the Results section. Linear correlation analysis was used to explore the correlation between blood loss and relevant factors, and was used to analyze the influence factors of operative time, exhaust time, and hospital stay. Multivariable linear regression analysis was used to detect the association between operative fever and related clinical characteristics. All tests were two-tailed, and *p* < 0.05 was considered statistically significant.

## Results

The selection process for this study population is presented in Figure 1. From our hospital, a total of 642 patients with uterine fibroid were initially recruited into this study. After excluding multiple fibroids, simultaneous other surgery, and choosing of hysterectomy/HIFU treatment, a total of 277 patients were available for the final analysis. The descriptive data of the study participants are shown in Table 1. The average age at recruitment was 38.09 ± 7.29 years, and the average BMI was 22.36 ± 2.92. Patients with diabetes and hypertension accounted for 2.2% and 2.5%, respectively. Prior pelvic surgery was performed in 44.8% of these patients, including cesarean section in 36.1%. The proportion of patients in the three groups was as follows: multiport laparoendoscopic surgery 93(33.6%), transumbilical laparoendoscopic single-site surgery (LESS) 90 (32.5%), and vNOTES 94 (33.9%).

**Table 1:** Description of the patients’ demographic characteristics and operation types.

Variables	Total
Patients	277
Age	38.09 ± 7.29
BMI (kg/m^2^)	22.36 ± 2.92
Max diameter of myoma	6.38 ± 1.59
Dysmenorrhea	72 (26.0%)
Diabetes	6 (2.2%)
Hypertension	7 (2.5%)
History of pelvic surgery	124 (44.8%)
Previous delivery mode	
Cesarean section	100 (36.1%)
Vaginal delivery	122 (44.0%)
Un-delivery	55 (19.9%)
Myomectomy type	
Multiport laparoscopy	93 (33.6%)
LESS	90 (32.5%)
vNOTES	94 (33.9%)

BMI, body mass index; LESS, laparoendoscopic single-site surgery; vNOTES, vaginal natural orifice transluminal endoscopic surgery.

Further analyzing the demographic data, there was no statistically significant differences between the three groups in terms of BMI, surgical history, parity, and maximum diameter of the fibroids. However, significant difference in age distribution was found. Older patients easily chose multiport laparoscopy, while younger patients were more likely to choose the single-port approach (Table 2) (*p* < 0.001) (Table 2).

**Table 2:** Description of the patient characteristics by myomectomy types.

Variables	Multiport	LESS	vNOTES	*p*-value
Patients	*N* = 93	*N* = 90	*N* = 94	
Age (year)	39.82 ± 6.24	36.60 ± 7.97	37.82 ± 7.30	0.010^a^
BMI (kg/m2)	22.54 ± 3.11	22.11 ± 2.84	22.42 ± 2.81	0.592^b^
Dysmenorrhea	16 (17.2%)	29 (32.2%)	27 (28.7%)	0.052^c^
History of pelvic surgery	41 (44.1%)	38 (42.2%)	45 (47.9%)	0.734^c^
Max diameter of myoma (cm)	6.09 ± 1.68	6.62 ± 1.59	6.44 ± 1.46	0.065^b^
Preoperative hemoglobin (g/L)	124.28 ± 17.75	125.84 ± 18.63	122.16 ± 18.37	0.453^b^
Gestation status				
Cesarean section	38 (40.9%)	26 (28.9%)	36 (38.3%)	0.208^c^
Vaginal delivery	42 (45.2%)	39 (43.3%)	41 (43.6%)	0.964^c^
Un-delivery	13 (14.0%)	25 (27.8%)	17 (18.1%)	0.351^c^
Operative information				
Procedure time (min)	80.82 ± 31.98	112.52 ± 42.05	106.80 ± 41.87	0.000^b^
Bleeding volume (ml)	62.37 ± 85.53	92.22 ± 131.05	78.09 ± 106.04	0.180^b^
Surgical conversion	0	0	2 (2.12%)	
Surgical injury	0	1 (bladder)	1 (rectum)	
Postoperative information				
Hemoglobin decline (g/L)	10.91 ± 5.11	12.11 ± 6.09	11.78 ± 6.00	0.780^b^
Hospital stay (day)	4.31 ± 1.36	4.08 ± 1.42	3.67 ± 1.78	0.016^b^
Exhaust time (hour)	37.14 ± 15.25	37.39 ± 14.31	31.52 ± 13.19	0.007^b^
Infection	1 (1.1%)	1 (1.1%)	7 (7.5%)	0.017^d^
Postoperative fever	38 (40.9%)	3 (6.7%)	25 (26.6%)	0.000^c^

BMI, body mass index; LESS, laparoendoscopic single-site surgery; vNOTES, vaginal natural orifice transluminal endoscopic surgery.

^a^ Average and standard deviation: Kruskal–Wallis test.

^b^ Average and standard deviation: One-way analysis of variance.

^c^ Number (%): χ^2^ test.

^d^ Number (%): Fisher’s exact test.

As Table 2 shows, there were significant differences in the time of surgery among the three groups. Multiple linear regression was used to analyze the effect of perioperative characteristics on the duration of surgery. After adjusting for tumor location, BMI, history of pelvic surgery, and abdominal/vaginal approach, the results revealed that operative time was positively correlated with the tumor size, multi/single-port, and pelvic adhesions. The operative time increased by 3.5 min when the tumor size increased by 1 cm (95% CI, 0.854–6.229, *p *= 0.01). Especially, switching from multiport laparoscopic to single-port laparoscopic surgery for the same size uterine fibroids, operative time would take 25 min longer (95% CI, 14.710–35.658, *p *< 0.000). The surgical time was prolonged by 10 min for each grade of pelvic adhesion (95% CI, 3.938–17.022, *p *= 0.002) (Table 3).

**Table 3:** Association between procedure time and myomectomy types.

Variables	Beta	95% CI	*p*-value
Tumor size	3.542	0.854 to 6.229	0.010
Tumor location	−0.019	−6.799 to 4.705	0.720
BMI	0.118	−1.284 to 1.521	0.868
History of pelvic surgery	−3.704	−12.444 to 5.037	0.405
History of cesarean section	9.080	−.010 to 18.169	0.050
Multi/single-port	25.184	14.710 to 35.658	<0.001
Abdominal/vaginal approach	−0.832	−11.058 to 9.394	0.873
Pelvic adhesions	10.480	3.938 to 17.022	0.002

BMI, body mass index.

To analyze the influencing factors of intraoperative hemorrhage, multivariable linear regression showed that operative bleeding was positively associated with tumor size (beta-value: 11.958, 95% CI, 4.286–19.630, *p* = 0.002) and operative time (beta-value: 1.117, 95% CI, 0.798–1.436, *p* < 0.001), and were not found to have a correlation with tumor location, BMI, history of surgery, myomectomy types (multi/single-port or abdominal/vaginal approach), or pelvic adhesion (Table 4). It was known that the length of surgical time was related to the operative type (multiport/single-port). If the operative type and surgical time were included in the study of the influencing factors of intraoperative bleeding, there might be mutual interference. Therefore, we included different variables and conducted multiple linear regression statistical analysis. The results showed that *R*^2^ was 0.489 when operative type and surgical time were included, and variance inflation factor of operative type and surgical time was 1.65 and 1.28, respectively. If either variable was excluded, *R*^2^ was <0.3, which means that statistical explanatory power decreased. Therefore, we suggest that the operative type and surgical time should be included in the analysis of intraoperative bleeding by multiple linear regression. In addition, there were five patients with intraoperative blood loss >500 ml, which was not included in statistical analysis due to large dispersion. Two of them were in the LESS group and three patients in the vNOTES group. There was no special cause of intraoperative bleeding, except that the uterine fibroids were all larger than 8 cm in size, and one patient had a fibroid size of 15 cm.

**Table 4:** Association between operative bleeding and myomectomy types.

Variables	Beta	95% CI	*p*-value
Tumor size	11.958	4.286 to 19.630	0.002
Tumor location	9.522	−6.942 to 25.987	0.256
BMI	2.695	−1.314 to 6.704	0.187
History of pelvic surgery	−0.216	−25.323 to 24.891	0.987
History of cesarean section	−5.595	−31.849 to 20.658	0.675
Multi/single-port	−17.894	−49.106 to 13.318	0.260
Abdominal/vaginal approach	−4.510	−33.842 to 24.822	0.762
Pelvic adhesions	−8.608	−27.695 to 10.478	0.375
Operative time	1.117	0.798 to 1.436	<0.001

BMI, body mass index.

About intraoperative complications, in 277 patients, 1 bladder injury occurred in the LESS group and 1 rectum injury in the vNOTES group. Pelvic adhesions were the cause of both cases. Another special condition was change in the surgical way during procedures. Two cases (2.12%) of surgical conversion occurred in the vNOTES group. In one case, the patient with intraoperative intestinal injury was changed to single-port laparoscopy for repairing the bowel. The other case was the first one of vNOTES myomectomy. To ensure safety, abdominal incision and endoscopy were used to monitor the procedure.

As Table 2 shows, the value of hemoglobin reduction in the multiport group was 10.91 ± 5.11 g/L, in the LESS group 12.11 ± 6.09 g/L, and that of the vNOTES group was 11.78 ± 6.00 g/L; the difference between the three groups was not statistically significant. The length of hospital stay in the Multiport, LESS and vNOTES groups were 4.31 ± 1.36, 4.08 ± 1.42, and 3.67 ± 1.78 days, respectively. The difference between the three groups was statistically significant.

**Figure 2:**
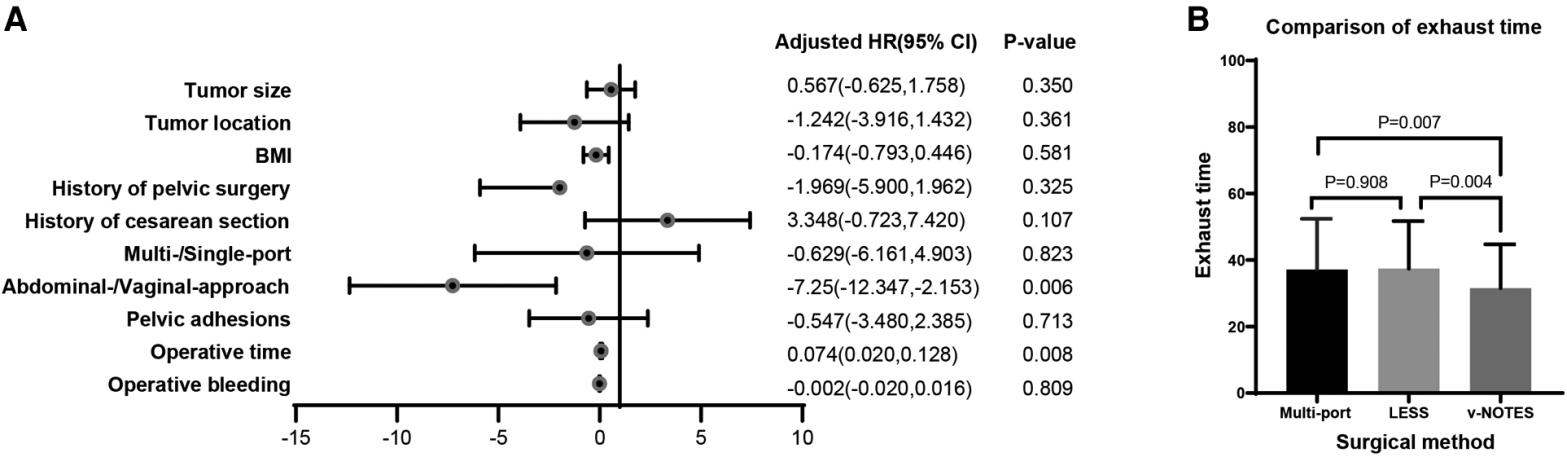
The impact of surgical characteristics on postoperative exhaust time. (**A**) Multivariable linear regression showed that postoperative exhaust time was positively associated with abdominal/vaginal approach type, with the postoperative exhaust time getting reduced by 7.25 h when the surgical approach is changed from transabdominal to transvaginal (beta-value: −7.250, 95% CI, −12.347 to −2.153, *p* = 0.006) and operative time (beta-value: 0.074, 95% CI, 0.020–0.128, *p* = 0.008); (**B**) The postoperative exhaust time was significantly different between the myomectomy types (vNOTES group vs. LESS group, *p* = 0.004; vNOTES group vs. multiport group, *p* = 0.007).

Multivariable linear regression showed that postoperative exhaust time was highly correlated with surgical approach (beta-value: −7.250, 95% CI, −12.347 to −2.153, *p* = 0.006). Another issue related to exhaustion is the duration of surgery (beta-value: 0.074, 95% CI, 0.020–0.128, *p* = 0.008) (Figures 2A,B) and were not found to have a correlation with tumor location, BMI, history of surgery, myomectomy types, or pelvic adhesion (Table 4).

We defined any oral temperature of 38.0°C or more occurring at 4 or more hours postoperatively as postoperative fever. The incidence of postoperative fever in the multiport group was 38 (40.9%), 3 (6.7%) in the LESS group, and 25 (26.6%) in the vNOTES group. There was statistically significant difference in the three groups. Noteworthy, 9 patients developed postoperative infection, with 7 (7.5%) from the vNOTES group (Table 2).

## Discussion

vNOTES surgery has become a novel type of gynecological micro-non-invasive technology which is prevalent all over the world. It has been proved as a more minimally invasive surgical approach for a variety of gynecological procedures, including ovarian cystectomy, salpingectomy, myomectomy, hysterectomy, and even early-stage cancer surgery (30–32). The advantages of vNOTES include less pain, faster recovery, and better hiding of surgical incisions for cosmetic effects (33). The purpose of this study was to comprehensively evaluate the advantages and disadvantages of traditional multiport laparoscopy, LESS, and vNOTES in uterine myomectomy, so as to provide a more theoretical basis for vNOTES surgery in a wider area in the future.

Previous studies focused on the feasibility of vNOTES in myomectomy (22, 34). Our study is the first to compare three minimally invasive fibroid removal techniques. No difference in the general conditions of patients in each group ensures demographic baseline homogeneity between groups. In the process of preoperative communication with patients, it was obvious that patients of different ages had various concerns. Older patients mostly only care about the approach difference between laparoscopy and laparotomy, and slight concern about difference in the number of ports in the abdomen. However, younger patients are more likely to choose single-port laparoscopy. But considering whether vaginal wounds affect pregnancy and delivery is unknown, and patients who have not delivered are more likely to choose single-port umbilical approach rather than transvaginal approach. Of course, our previous study has shown that vNOTES surgery does not affect subsequent pregnancy and delivery, where more data are needed to prove this (35).

There were significant differences in the operative time among the three groups. Except for the correlation with the two inherent variables (fibroids size and pelvic adhesion), the operative time was significantly correlated with the number of ports. Whether single-port surgery is through the umbilicus or the vagina, the operative time is more than the multiport laparoscopy. Under the same conditions, the time of single-port laparoscopic surgery was extended by 25 min. The main reason is the difficulty of single-port laparoscopy, such as mutual interference between instruments, no assistant's help, passive position of the surgeon, etc. (36–38). However, the overall time extension is not very much, being within the acceptable range. It is worth mentioning that the introduction of robotic surgery, thanks to the instruments articulation and the precision of movement, made possible to improve these difficulties (39, 40). In addition, single pore has the advantage of safe and effective specimen removal, avoiding tumor spread caused by morcellation (41, 42). The advantage of vNOTES in specimen removal is much more significant, mainly because the vaginal wall is more elastic than the navel, and less dependent on muscle relaxants.

Another information of concern is the intraoperative blood loss. Our data showed that there were differences in intraoperative bleeding among the three groups, but this diversity was caused by the size of uterine fibroids and the time of surgery, not by the surgical approach. Five patients with intraoperative blood loss >500 ml were not included in statistical analysis due to large dispersion. We analyzed these five patients separately. Two of them were in the LESS group and three in the vNOTES group. There was no special cause of intraoperative bleeding, except that the uterine fibroids were all larger than 8 cm in size, and even one patient had a fibroid of 15 cm. Previous studies have shown that when the diameter of fibroid was relatively large, the blood loss in the multiport group was less than that of the LESS group (43, 44). Despite the excessive bleeding, there was no transfer to other operations and the surgery was continued and completed safely.

In our study, two patients in the vNOTES group underwent two surgical approaches. One case was the first vNOTES myomectomy performed in our hospital. To ensure the safety and feasibility of the operation, the surgery was performed with an umbilical endoscopic monitor. The second patient had rectal injury and was transferred to transumbilical single-port laparoscopy for rectal repair. This patient was discharged successfully after *in situ* repair without fistula. The 2.12% intraoperative conversion rate is consistent with previous findings (45, 46). The major complication of vNOTES surgery is injury to adjacent organs, especially the rectum, and injury occurs mostly during the establishment of the operating platform. Our experience included a careful inquiry about the history of dysmenorrhea and a physical examination to assess uterine mobility and tenderness of tubercles on the surface of the sacro ligament. Vaginal ultrasound can also be used to assess the sliding of the uterus on the anterior wall of the rectum in real time.

Then, compared with the postoperative recovery of patients in the three groups, vNOTES had obvious advantages in postoperative exhaust time. Thus, patients required shorter postoperative hospitalization time and less expense. The main reasons for early postoperative exhaust time are as follows. First, vNOTES surgery is basically performed in the pelvic cavity and has little influence on the upper abdomen. Second, the intestine was pushed up to the level of the true pelvis before surgery, so that the surgical instruments would not repeatedly touch the intestinal canal. This would reduce the stimulation of the intestinal tract. Moreover, since the surgical perspective is upward, the blood gathers between the endoscopic body and the target area, and so the blood should be washed continuously during the operation to ensure a clear visual field. As a result, the postoperative residual blood volume in the abdominal cavity is significantly reduced, resulting in less chemical stimulation, less release of inflammatory factors, and faster recovery of intestinal function. Finally, the vNOTES platform is based on the posterior vaginal fornix, where visceral nerves are distributed mainly. It is different from the abdominal platform that damages the somatosensory nerves. Thus, vNOTES patients have less postoperative pain, which is beneficial for them to get out of bed and promote intestinal peristalsis more quickly.

Despite these advantages, our study found an increased risk of infection after the vNOTES procedure, which is inconsistent with previous studies (47–49). Pelvic infection occurred in seven patients in the vNOTES group. It is a too small number to be statistically analyzed, but the reason was definitely related to the approach of the surgery. This suggests that patients receiving vNOTES need to use antibiotics during the perioperative period and be more strict in preoperative vaginal disinfection. Meanwhile, It should be noted that vNOTES is not applicable to all myomectomies. For fibroids at the bottom of the uterus or broad ligament, vNOTES is not applicable due to the limitations of its field of vision and surgical instrument scope. Our study found that if the fibroids are too low to be stably placed, ports are also not suitable for this technique. For multiple uterine fibroids located simultaneously in the anterior and posterior walls of the uterus, due to the need for two approaches to open the anterior and posterior vaults separately, more trauma caused and manipulation steps are cumbersome, so it is not included in the scope of myomectomy with vNOTES. In addition to the fibroids located at the above-mentioned special site, this technique is not suitable for patients with severe endometriosis, previous pelvic infection, and uncured vaginitis. However, obesity and history of pelvic surgery are not contraindications for this approach.

The merits of our study are the specialized study population and the standard operating procedures of vNOTES. The participants were screened using strict inclusion and exclusion criteria for this retrospective study. Patients with multiple uterine fibroids were excluded. According to the postoperation pathological report, patients with adenomyoma were also excluded. Additionally, all patients underwent outpatient review 1 month after surgery to check postoperative recovery and complete clinical data, resulting in a comprehensive study design. In addition, our hospital has carried out vNOTES since 2018; the number of vNOTES is nearly 2000 cases per year in recent two years, and now there is quite mature experience and standard operating procedures of vNOTES.

This preliminary study adds to our understanding of multiport, transumbilical single-site, and transvaginal natural orifice endoscopic surgery for myomectomy; however, it has some limitations that should be considered. First, the sample size in this study was relatively modest compared with similar studies on multiport and TU-LESS. Second, this study is a retrospective study and vNOTES has been used in gynecology only for 5 years. Prospective follow-up of women after myomectomy can more clearly clarify their short- and long-term complications, as well as the potential effects of vNOTES on patients’ sexual function, pregnancy, and vaginal delivery. To achieve this goal, a large-scale study involving more patients and different types of gynecological diseases with vNOTES conducted in multiple centers is required.

## Conclusions

Therefore, our study confirms that vNOTES is equally safe and effective in uterine myomectomy compared with multiport and single-port laparoscopy, and can be used as a routine procedure for patients. vNOTES patients recover faster after surgery, but still attention should be paid to preoperative evaluation, strengthen preoperative disinfection and vaginal preparation, timely conversion during surgery, call experienced doctors on stage, and routine use of antibiotics to prevent postoperative infection.

## Data Availability

The raw data supporting the conclusions of this article will be made available by the authors, without undue reservation.

## References

[1] Parker WH. Etiology, symptomatology, and diagnosis of uterine myomas. Fertil Steril. (2007) 87(4):725–36. 10.1016/j.fertnstert.2007.01.093 17430732

[2] Jain N Kriplani I Sharma S Hanumantaiya S Kriplani A. Urinary retention unveiling deeply embedded multiple leiomyomas in women with Mayer-Rokitansky-Kuster-Hauser syndrome and its successful laparoscopic management: a case-report and literature review. J Surg Case Rep. (2022) 2022(6):rjac291. 10.1093/jscr/rjac291 35721263 PMC9202637

[3] Sparic R Mirkovic L Malvasi A Tinelli A. Epidemiology of uterine myomas: a review. Int J Fertil Steril. (2016) 9(4):424–35. 10.22074/ijfs.2015.4599 26985330 PMC4793163

[4] Mlodawska OW Saini P Parker JB Wei J-J Bulun SE Simon MA Epigenomic and enhancer dysregulation in uterine leiomyomas. Hum Reprod Update. (2022) 28(4):518–47. 10.1093/humupd/dmac008 35199155 PMC9247409

[5] Zhai YH Zheng Z Deng W Yin J Bai Z-G Liu X-Y Inflammation-related indicators to distinguish between gastric stromal tumors and leiomyomas: a retrospective study. World J Clin Cases. (2022) 10(2):458–68. 10.12998/wjcc.v10.i2.458 35097070 PMC8771401

[6] DiMauro A Seger C Minor B Amitrano AM Okeke I Taya M Prolactin is expressed in uterine leiomyomas and promotes signaling and fibrosis in myometrial cells. Reprod Sci. (2022) 29(9):2525–35. 10.1007/s43032-021-00741-w 34724171

[7] Grcevich LO O’Connell A Jabaay MJ Scott JT. Postmenopausal uterine leiomyomas and chronic lymphadenopathy: exploring epigenetic changes and pathophysiology. Cureus. (2021) 13(9):e18274. 10.7759/cureus.18274 34722052 PMC8545538

[8] Simon A Robinson F Anzivino A Boyer M Hendricks-enger A Guilliams D Histotripsy for the treatment of uterine leiomyomas: a feasibility study in ex vivo uterine fibroids. Ultrasound Med Biol. (2022) 48(8):1652–62. 10.1016/j.ultrasmedbio.2022.04.214 35641394

[9] Bajaj S Gopal N Clingan MJ Bhatt S. A pictorial review of ultrasonography of the FIGO classification for uterine leiomyomas. Abdom Radiol (NY). (2022) 47(1):341–51. 10.1007/s00261-021-03283-6 34581926

[10] Gursoy AY Cakmak D Akgul G Kiseli M Umudum H Pabuccu R The assessment of the relationship between the vascularity of FIGO type 4-7 leiomyomas and abnormal uterine bleeding. J Obstet Gynaecol. (2022) 42(1):153–7. 10.1080/01443615.2021.1882969 33938365

[11] Giuliani E As-Sanie S Marsh EE. Epidemiology and management of uterine fibroids. Int J Gynaecol Obstet. (2020) 149(1):3–9. 10.1002/ijgo.13102 31960950

[12] Zheng Y Chen L Liu M Wu J Yu R Lv F. Prediction of clinical outcome for high-intensity focused ultrasound ablation of uterine leiomyomas using multiparametric MRI radiomics-based machine leaning model. Front Oncol. (2021) 11:618604. 10.3389/fonc.2021.618604 34567999 PMC8461183

[13] Zheng Y Chen L Liu M Wu J Yu R Lv F. Nonenhanced MRI-based radiomics model for preoperative prediction of nonperfused volume ratio for high-intensity focused ultrasound ablation of uterine leiomyomas. Int J Hyperthermia. (2021) 38(1):1349–58. 10.1080/02656736.2021.1972170 34486913

[14] Ergul A. Quality and reliability of YouTube videos on surgical treatment of uterine leiomyomas. Cureus. (2021) 13(11):e20044. 10.7759/cureus.20044 34987925 PMC8717934

[15] Hoshiai H Seki Y Kusumoto T Kudou K Tanimoto M. Relugolix for oral treatment of uterine leiomyomas: a dose-finding, randomized, controlled trial. BMC Womens Health. (2021) 21(1):375. 10.1186/s12905-021-01475-2 34711224 PMC8555132

[16] Panesar H Dhaliwal HS. Iatrogenic parasitic leiomyomas: a late and uncommon complication after laparoscopic morcellation. Cureus. (2022) 14(5):e24718 10.7759/cureus.24718. 35676984 PMC9166603

[17] Rosati A Fedele C Fagotti A Lafuenti L Gioè A Chieffo DPR Needleoscopic-assisted risk-reducing bilateral salpingo-oophorectomy in BRCA1/2 mutation carriers: peri-operative outcomes and psychological impact. Eur J Obstet Gynecol Reprod Biol. (2022) 273:1–6. 10.1016/j.ejogrb.2022.03.040 35429923

[18] Noh JJ Kim MS Kang JH Jung J-H Chang C-S Jeon J Comparison of surgical outcomes of hysterectomy by vaginal natural orifice transluminal endoscopic surgery (vNOTES) versus single-port access (SPA) surgery. J Pers Med. (2022) 12(6):875. 10.3390/jpm12060875 PMC9225248 35743660

[19] Jegaden M Debras E Pourcelot AG Capmas P Fernandez H. vNOTES for ovarian drilling: a new minimal invasive technique. J Minim Invasive Gynecol. (2022) 29(8):932–33. 10.1016/j.jmig.2022.06.007 35697286

[20] Sunkara S Guan X. Robotic vaginal natural orifice transluminal endoscopic myomectomy. Fertil Steril. (2022) 118(2):414–6. 10.1016/j.fertnstert.2022.05.009 35691722

[21] Baekelandt J. Transvaginal natural-orifice transluminal endoscopic surgery: a new approach to myomectomy. Fertil Steril. (2018) 109(1):179. 10.1016/j.fertnstert.2017.09.009 29129378

[22] Badiglian-Filho L Fukazawa EM Faloppa CC Baiocchi G. VNOTES (Vaginal Natural Orifices Transluminal Endoscopic Surgery) myomectomy through anterior cul-de-sac approach on the bicornuate uterus. J Gynecol Obstet Hum Reprod. (2021) 50(1):101911. 10.1016/j.jogoh.2020.101911 32931956

[23] Musselman K Laurence J Magro C Rahbari P Di Vitantonio T Havryliuk Y. Atypical hemolytic uremic syndrome after myomectomy: a case report. Case Rep Womens Health. (2022) 35:e00424. 10.1016/j.crwh.2022.e00424 35769946 PMC9234065

[24] Lagana AS Vitagliano A Casarin J Garzon S Uccella S Franchi M Transvaginal versus port-site specimen retrieval after laparoscopic myomectomy: a systematic review and meta-analysis. Gynecol Obstet Invest. (2022) 87(3–4):177–83. 10.1159/000525624 35728574

[25] Ruiz de Santaquiteria Torres V Palomo Lopez R Rubio Arroyo M Alemán Mahechac NF Iglesiasd DLL Agurto Rivera SN Carbon monoxide poisoning and air embolism following hysteroscopic myomectomy: a case report. J Gynecol Obstet Hum Reprod. (2022) 51(8):102431. 10.1016/j.jogoh.2022.102431 35718331

[26] Dildy GA 3rd Paine AR George NC Velasco C. Estimating blood loss: can teaching significantly improve visual estimation? Obstet Gynecol. (2004) 104(3):601–6. 10.1097/01.AOG.0000137873.07820.34 15339775

[27] Rybak EA Polotsky AJ Woreta T Hailpern SM Bristow RE. Explained compared with unexplained fever in postoperative myomectomy and hysterectomy patients. Obstet Gynecol. (2008) 111(5):1137–42. 10.1097/AOG.0b013e31816baea8 18448746

[28] Lewis TL Mason L Ray R. The Clavien-Dindo complication classification modified for foot and ankle orthopaedic surgery. Foot Ankle Surg. (2022) 28(6):800–2. 10.1016/j.fas.2022.03.006. 35346593

[29] Murphy DJ Stirrat GM Heron J Team AS. The relationship between Caesarean section and subfertility in a population-based sample of 14 541 pregnancies. Hum Reprod. (2002) 17(7):1914–7. 10.1093/humrep/17.7.1914 12093860

[30] Yoshiki N. Review of transvaginal natural orifice transluminal endoscopic surgery in gynecology. Gynecol Minim Invasive Ther. (2017) 6(1):1–5. 10.1016/j.gmit.2016.11.007 30254860 PMC6113962

[31] Huang L Lin YH Yang Y Gong ZL He L. Comparative analysis of vaginal natural orifice transluminal endoscopic surgery versus transumbilical laparoendoscopic single-site surgery in ovarian cystectomy. J Obstet Gynaecol Res. (2021) 47(2):757–64. 10.1111/jog.14603 33331001

[32] Jung Y Rattanaburi A Kim O Park JH Lee KH. A simple gasless direct suturing technique to achieve ovarian hemostasis during transvaginal natural orifice transluminal endoscopic surgery ovarian cystectomy. J Laparoendosc Adv Surg Tech A. (2021) 31(9):1046–50. 10.1089/lap.2020.0575 33121358

[33] Park SJ Kim HS Yim GW. Comparison of vaginal natural orifice transluminal endoscopic surgery (vNOTES) and laparoendoscopic single-site (LESS) hysterectomy on postoperative pain reduction: a randomized pilot study. Pain Ther. (2021) 10(2):1401–11. 10.1007/s40122-021-00300-w 34374960 PMC8586123

[34] Liu J Lin Q Blazek K Liang B Guan X. Transvaginal natural orifice transluminal endoscopic surgery myomectomy: a novel route for uterine myoma removal. J Minim Invasive Gynecol. (2018) 25(6):959–60. 10.1016/j.jmig.2018.01.011 29410143

[35] Feng D He L. Pregnancy and childbirth after transvaginal natural orifice transluminal endoscopic surgery for benign gynecological diseases. Int J Gynaecol Obstet. (2021) 155(3):551–2. 10.1002/ijgo.13820 34241832

[36] Baekelandt J Noori N Hofmann L Mansoor A Kapurubandara S. Standardised step by step approach to adnexectomy by Vaginal Natural Orifice Transluminal Endoscopic Surgery. Eur J Obstet Gynecol Reprod Biol. (2022) 274:160–5. 10.1016/j.ejogrb.2022.05.021 35653905

[37] Huber D Hurni Y. Sentinel node biopsy for endometrial cancer by retroperitoneal transvaginal natural orifice transluminal endoscopic surgery: a preliminary study. Front Surg. (2022) 9:907548. 10.3389/fsurg.2022.907548 35615644 PMC9125023

[38] Badiglian-Filho L Baiocchi G Baekelandt J. 10 steps to approach large ovarian masses through vNOTES. Int J Gynecol Cancer. (2022) :ijgc-2022-003421. 10.1136/ijgc-2022-003421. [Epub ahead of print] 35580920

[39] Capozzi VA Armano G Rosati A Tropea A Biondi A. The robotic single-port platform for gynecologic surgery: a systematic review of the literature and meta-analysis. Updates Surg. (2021) 73(3):1155–67. 10.1007/s13304-020-00812-8 32472402

[40] Rumolo V Rosati A Tropea A Biondi A Scambia G. Senhance robotic platform for gynecologic surgery: a review of literature. Updates Surg. (2019) 71(3):419–27. 10.1007/s13304-018-00620-1 30659479

[41] Kita M Sumi G Butsuhara Y Hisamatsu Y Okada H. Resection of vaginal recurrence of granulosa cell tumor by pneumovaginal endoscopic surgery. Gynecol Oncol Rep. (2021) 36:100743. 10.1016/j.gore.2021.100743 33748384 PMC7970270

[42] Badiglian-Filho L Fukazawa EM Faloppa C Baiocchi G. Ovarian sparing cystectomy for borderline serous tumor through vNOTES (vaginal Natural Orifices Transluminal Endoscopic Surgery). Int J Gynecol Cancer. (2020) 30(8):1253–4. 10.1136/ijgc-2020-001513 32624499

[43] Wu PC Sheu BC Huang KJ Huang SC Chang WC. Laparoendoscopic two-site myomectomy (LETS-M) using conventional laparoscopic instruments and the glove-port technique. J Formos Med Assoc. (2022) 121(11):2248–56. 10.1016/j.jfma.2022.04.013 35570051

[44] Lagana AS Garzon S Dababou S Uccella Sb Medvediev Mc Pokrovenko Dc Prevalence of intrauterine adhesions after myomectomy: a prospective multicenter observational study. Gynecol Obstet Invest. (2022) 87(1):62–9. 10.1159/000522583 35168241

[45] Liu J Tan L Thigpen B Koythong T Zhou X Liu Q Evaluation of the learning curve and safety outcomes in robotic assisted vaginal natural orifice transluminal endoscopic hysterectomy: a case series of 84 patients. Int J Med Robot. (2022) 18(3):e2385. 10.1002/rcs.2385 35236012

[46] Ozceltik G Hortu I Itil IM Yeniel AO. Vaginal approach versus laparoscopy for hysterectomy in transgender men. J Gynecol Obstet Hum Reprod. (2022) 51(2):102286. 10.1016/j.jogoh.2021.102286 34910989

[47] Karkia R Giacchino T Taylor J Ghaffar A Gupta A Kovoor E. Hysterectomy and adenextomy via transvaginal natural orifice transluminal endoscopic surgery (vNOTES): a UK perspective with a case series of 33 patients. Eur J Obstet Gynecol Reprod Biol. (2019) 242:29–32. 10.1016/j.ejogrb.2019.08.023 31539766

[48] Liu J Kohn J Fu H Guan Z Guan X. Transvaginal natural orifice transluminal endoscopic surgery for sacrocolpopexy: a pilot study of 26 cases. J Minim Invasive Gynecol. (2019) 26(4):748–53. 10.1016/j.jmig.2018.08.009 30165180

[49] Baekelandt JF De Mulder PA Le Roy I Mathieu C Laenen A Enzlin P Transvaginal natural orifice transluminal endoscopic surgery (vNOTES) adnexectomy for benign pathology compared with laparoscopic excision (NOTABLE): a protocol for a randomised controlled trial. BMJ Open. (2018) 8(1):e018059. 10.1136/bmjopen-2017-018059 29326183 PMC5780723

